# ANATOMICAL VARIATIONS OF THE SUPERIOR MESENTERIC ARTERY AND ITS CLINICAL AND SURGICAL IMPLICATIONS IN HUMANS

**DOI:** 10.1590/0102-672020190001e1508

**Published:** 2020-08-24

**Authors:** Natasha Gabriela Oliveira da SILVA, Ana Beatriz Marques BARBOSA, Nathalie de Almeida SILVA, Diego Neves ARAÚJO, Thiago de Oliveira ASSIS

**Affiliations:** 1 Unifacisa University Center, Campina Grande, PB, Brazil; 2 Department of Biology, Paraíba State University, Campina Grande, PB, Brazil; 3 Academic Unit of Medical Sciences, Federal University of Campina Grande, Campina Grande, PB, Brazil

**Keywords:** Mesenteric artery, superior, Anatomy, Intestinal atresia, Intestinal obstruction, Anatomic variation, Artéria mesentérica superior, Anatomia, Atresia intestinal, Obstrução intestinal, Variação anatômica

## Abstract

**Introduction:**

Superior mesenteric artery (SMA) usually arises from the abdominal aorta, just below the celiac trunk and it supplies the midgut-derived embryonic structures. Anatomical variations in this vessel contribute to problems in the formation and/or absorption of this part of the intestine and its absence has been recognized as the cause of congenital duodenojejunal atresia.

**Objective:**

To analyze SMA anatomical variations in humans and the possible associated clinical and surgical implications.

**Methods:**

This is a systematic review of papers indexed in PubMed, SciELO, Springerlink, Science Direct, Lilacs, and Latindex databases. The search was performed by two independent reviewers between September and December 2018. Original studies involving SMA variations in humans were included. SMA presence/absence, level, place of origin and its terminal branches were considered.

**Results:**

At the end of the search, 18 studies were selected, characterized as for the sample, method to evaluate the anatomical structure and main results. The most common type of variation was when SMA originated from the right hepatic artery (6.13%). Two studies (11.11%) evidenced the inferior mesenteric artery originating from the SMA, whereas other two (11.11%) found the SMA sharing the same origin of the celiac trunk.

**Conclusion:**

SMA variations are not uncommon findings and their reports evidenced through the scientific literature demonstrate a great role for the development of important clinical conditions, making knowledge about this subject relevant to surgeons and professionals working in this area.

## Graphical Abstract

[Fig f1a]

**Figure f1a:**
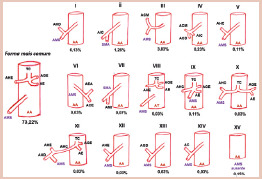
Different morphological patterns of SMA

## RESUMO

**Introdução:**

A artéria mesentérica superior (AMS), normalmente, tem sua origem a partir da aorta abdominal, um pouco abaixo do tronco celíaco e é responsável pela irrigação das estruturas derivadas, embrionariamente, do intestino médio. Variações anatômicas nesse vaso contribui para defeitos na formação e/ou absorção dessa parte do intestino e a sua ausência tem sido reconhecida como a causa da atresia duodenojejunal congênita.

**Objetivo:**

Analisar as variações anatômicas dela em humanos e as possíveis implicações clínicas e cirúrgicas associadas.

**Métodos:**

Trata-se de uma revisão sistemática de artigos indexados nas bases de dados PubMed, SciELO, Springerlink, Scienc Direct, Lilacs e Latindex. A busca ocorreu por dois revisores independentes entre setembro e dezembro de 2018. Foram incluídos artigos originais envolvendo as variações da AMS em humanos. Considerou-se para este estudo a presença/ausência da AMS, o nível, local de origem e seus ramos terminais.

**Resultados:**

Ao final da busca foram selecionados 18 artigos, caracterizados quanto à amostra, método para avaliar a estrutura anatômica e principais resultados. O tipo de variação mais comum foi aquele cuja AMS se originou da artéria hepática direita (6,13%). Dois estudos (11,11%) evidenciaram a artéria mesentérica inferior originando-se a partir da AMS, enquanto outros dois (11,11%) constataram ser ela compartilhada na mesma origem do tronco celíaco.

**Conclusão:**

Variações na AMS não são achados incomuns e seus relatos evidenciados através da literatura científica demonstram grande importância para o desenvolvimento de condições clínicas importantes, tornando o conhecimento sobre esse assunto relevante para os cirurgiões e profissionais atuantes nesta área.

## Graphical Abstract

[Fig f1as]

**Figure f1as:**
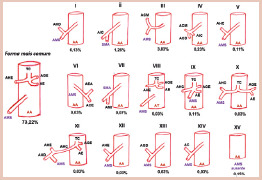
Diferentes padrões morfológicos da AMS.

## INTRODUCTION

The superior mesenteric artery (SMA) arises, classically, in the anterior part of the aorta and it is located 1 cm below the celiac trunk, posteriorly to the pancreas body and the splenic vein, at the level of intervertebral discs between L1 and L2, then going into the mesentery^20^.

This vessel arises from the aorta through the left renal vein and it supplies part of the small intestine, cecum, ascending colon and 2/3 of proximal transverse colon. Together with the inferior mesenteric artery and celiac trunk, SMA contributes to the vascularization of the gastrointestinal tract^7^.

SMA originates the middle colic, right colic, ileocolic, jejunal, ileal and appendicular arteries. Although this is commonly the classical anatomical pattern, some changes have been observed regarding the SMA branches, level and its origin. Such variations and their relationship with the surrounding structures are, therefore, important from a clinical and surgical perspective^17^ ^,^ ^20^.

In a study with 607 kidney donors and trauma patients, it was observed that 388 (63.9%) had a classic arterial pattern, whereas 219 (36.1%) presented some type of variation. Among the observed changes, one variation was more common than others, in which the SMA originated the right hepatic artery in 58 (9.6%) of the cases^2^.

Variations in the anatomy of this vessel may be related to the development of important clinical conditions, such as congenital duodenojejunal atresia, since SMA absence has been recognized as one of its causes in newborns. SMA absence contributes to problems in midgut formation or absorption. Patients with this type of variation are subject to death with no chance of surgical intervention^23^ ^,^ ^24^ ^,^ ^25^.

In this context, knowledge about these variations are relevant, considering that their study and investigation are important and valid, mainly for surgeons and professionals who work in this area, thus avoiding complications and iatrogenic situations.

This study aims to analyze the anatomical variations of SMA in humans and its possible clinical and surgical implications.

## METHOD

This is a systematic review. In order to conduct this study, the following databases were consulted: SciELO (Scientific Electronic Library Online); Springerlink; Science Direct; Pubmed (National Library of Medicine); Lilacs (Latin American and Caribbean Literature in Health Sciences) and Latindex. The research strategy involved such databases and their respective search terms: in SciELO and Springerlink: “Superior mesenteric artery” AND “Anatomy” AND “Anatomical variation.” In Lilacs, Latindex and Science Direct databases: “Superior Mesenteric Artery” AND “Absence of Superior Mesenteric Artery” AND “Anatomical Variation.” Whereas in Pubmed the following keywords were used: “Superior mesenteric artery” AND “Anatomical variation” AND “Absence of superior mesenteric artery”. The electronic search was performed by two independent reviewers between September and December 2018.

Were included original studies involving SMA in humans or studies on human cadavers. Reviews were excluded as well as those studies involving animals.

Studies found in more than one of the databases were counted only once. The selected papers were published between 2002 and 2016. In SCIELO, 18 studies were found, 1,182 in Springerlink, 831 in Science Direct, 56 in Lilacs, 275 in Pubmed and 0 in Latindex, totaling 2,362 papers. After abstract screening, the inclusion and exclusion criteria were applied, and 18 papers were selected for analysis.

The selected studies were critically analyzed by an interpretation guide, used to evaluate their individual quality, based on the studies of Greehalgh^8^ and adapted by Mcdermid et al.^15^. The studies quality evaluation items are expressed by scores in Table 1, in which 0=absent; 1=incomplete; and 2=complete.

### Statistical analysis

The search was performed by two independent reviewers, and the interobserver agreement analysis was performed using the Kappa test, using Prism V 5.0 software, according to Landis and Koch^14^ method. The value found was K=0.77 (substantial agreement).

## RESULTS

Table 1 shows the quality analysis of the selected studies for this study.

**TABLE 1:** Quality analysis of studies on SMA variations in humans

STUDIES	EVALUATION CRITERIA												
	1	2	3	4	5	6	7	8	9	10	11	12	Total (%)
Farghadani et al. (2016)	2	1	2	2	2	NA	2	2	2	2	2	2	95.45
Fonseca Neto et al. (2017)	1	2	2	1	2	NA	1	0	2	2	1	1	68.18
Gamo et al. (2016)	2	2	2	2	2	NA	2	2	2	2	2	1	95.45
Gomes et al. (2014)	1	NA	1	0	1	NA	0	0	0	1	1	0	25.00
Jain e Motwani et al. (2013)	2	0	2	1	1	NA	2	2	1	2	2	2	77.27
Kitamura et al. (1987)	1	NA	2	1	1	NA	0	0	1	2	0	0	40.00
Koops et al. (2004)	2	2	2	2	2	NA	2	2	2	1	2	1	90.90
Matusz et al. (2013)	2	NA	2	1	1	NA	2	0	2	2	2	2	80.00
Olave et al. (2009)	2	1	1	2	1	NA	1	1	2	2	1	1	68.18
Olga et al. (2010)	2	1	2	2	2	NA	2	2	2	2	2	2	95.45
Saša et al. (2016)	2	NA	2	0	1	NA	1	1	2	1	0	1	60.00
Sebben et al. (2013)	2	1	2	1	1	NA	1	1	2	2	2	1	72.72
Taha et al. (2017)	2	NA	1	0	1	NA	0	0	1	1	0	2	40.00
Torres et al. (1999)	0	NA	1	0	1	NA	1	0	0	1	0	0	20.00
Weber e Freeman (1999)	2	NA	1	0	1	NA	1	0	0	0	0	1	30.00
Wu et al. (2014)	2	NA	2	1	1	NA	1	1	2	0	0	1	55.00
Yakura et al. (2017)	1	NA	2	0	1	NA	1	0	2	1	0	1	45.00
Yoo et al. (2011)	2	NA	2	0	1	NA	0	0	2	1	1	1	50.00

NA = not applicable; evaluation criteria = 1. thorough literature review to define the research question; 2. specific inclusion/exclusion criteria; 3. specific hypotheses; 4. appropriate range of psychometric properties; 5. sample size; 6. follow up; 7. the authors refer to specific procedures for administration, scoring and interpretation of procedures; 8. measurement techniques have been standardized; 9. data were presented for each hypothesis; 10. appropriate statistics - point estimates; 11. appropriate statistical error estimates; 12. valid conclusions and clinical recommendations.

**FIGURE 1:**
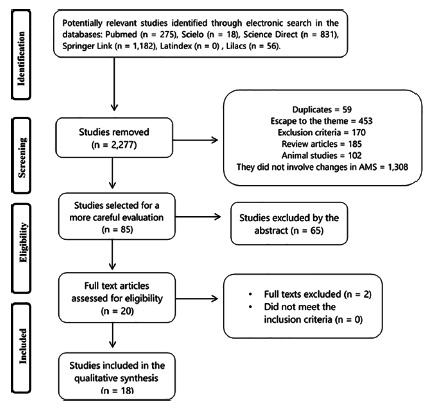
Studies included and excluded in the review on anatomical variations of the superior mesenteric artery

A summary of the electronic search in the selected databases is presented in Figure 1. Initially, 2,362 studies were identified, and 2,277 were removed because they did not have relevant data, changed the topic or because they were in duplicates, with 85 remaining, which were submitted to content analysis and verification of inclusion and exclusion criteria. Of these, 20 were read in full, and only 18 studies^2,3,5,6,9,11,12,16,18,13,21,22,23,24,25,26,27,28^ adequately fulfilled all inclusion criteria and were selected for this review.

Table 2 summarizes the selected studies for the analysis of the findings related to SMA.

**TABLE 2:** Characteristics of the studies that evaluated the anatomical variations in SMA

Author (year)	Sample	Methods	Main Results
Farghadani et al. (2016)	607 patients	Computed tomography	Three hundred and eighty-eight (63.9%) of the 607 patients had classic SMA anatomy and 219 (36.1%) had variant types, the most common type was the one from the right hepatic artery (9.6%).
Fonseca Neto et al. (2017)	479 patients	Vascular analysis of deceased liver donors	Four hundred and sixteen patients (86.84%) had normal arterial anatomy. The other 63 patients (13.15%) presented anatomical variation. Of these, 27 presented the superior mesenteric artery originating the right hepatic artery, whereas other 4 presented the right hepatic artery resulting from the superior mesenteric artery while the left hepatic artery originated from the left gastric artery.
Gamo. et al. (2016)	Sample #1: 28 men and 22 women (cadavers); Sample #2: 399 men and 161 women (alive)	Human cadaveric dissection and computed tomography	The variations found were classified into two types. In type I, SMA originated the middle colic artery (MCA), right colic (RCA) and ileocolical (ICA) in 40% of cadavers dissected and 73.69% of the CT scan (computed tomography). In type II, there are three distinct patterns: in the IIA, ICA arises separately (found in 20% of the dissected cadavers and 4.28% of the CT sample), in IIB the MCA is the one that arises separately (found in 32% of the cadavers dissected and 15% of the CT sample) and in the IIC, MCA, RCA and ICA appear from the common trunk (present in 0.35% of CTs and absent in cadavers).
Gomes et al. (2014)	One male cadaver	Cadaveric dissection	The common hepatic artery originated from the superior mesenteric artery, located 3.5 cm below and lateral to the celiac trunk, forming a hepatomesenteric trunk.
Jain and Motwani (2013)	20 cadavers	Cadaveric dissection	14 cadavers (70%) presented a normal SMA branch pattern, 5 cadavers (25%) had a common trunk for the ileocolic and right colonic arteries coming out of SMA, while 1 cadaver (5%) presented the rarest variation in the pattern of SMA branching: a common trunk of the left colonic artery with an accessory splenic artery arising from its anterior face, rather than from the inferior mesenteric artery.
Kitamura et al. (1987)	A 69-year-old Japanese cadaver	Cadaveric dissection	SMA originated the inferior mesenteric artery, which usually originates from the abdominal aorta. And although it emerged from SMA, it had the same branches as a lower mesenteric artery.
Koops et al. (2004)	604 patients	Analysis of superior celiac and mesenteric angiograms	The arterial anatomy considered normal in the literature was found in 79.1% of the exams. The aberrant right or accessory hepatic artery (RHA) branched out from the superior mesenteric artery in 11.9% of the cases.
Matusz et al. (2013)	A 44-year-old man	Computed tomography angiotomography	The celiac trunk and the superior mesenteric artery originate from the thoracic aorta (TA) 21 mm and 9 mm above the aortic hiatus, respectively. The SMA descends both at the thoracic and abdominal level, making a 17º angle, and having an aortomesenteric distance of 9 mm at the level of the third part of the duodenum.
Olave et al. (2009)	31 Chileanpatients, adults	Helical computed tomography	The superior mesenteric artery was found in 100% of the cases. The level of origin was always cranial to the origin of the renal arteries. The level of origin of the superior mesenteric artery was observed compared to LI vertebra in 16 cases and in the L2 vertebra in 8 cases.
Kornafel et al. (2010)	201 patients (91 womenand 110 men)	Computed tomography angiography	In 88 patients (43.8%), there were anatomical variations of the branched arteries from the abdominal aorta, including superior mesenteric artery variations in 4 (2%) patients. The common origin of the celiac trunk and the superior mesenteric artery - the celiac-mesenteric trunk - was observed in 3 patients (1.5%). The simultaneous presence of the gastroesplenic trunk and the hepatomesenteric trunk was found in 1 patient (0.5%).
Saša et al. (2016)	A prematureinfant (29 weeks)	Ultrasonographyand Abdominal Radiography	Investigation of the abdominal cavity revealed duodenal atresia in the second portion of the duodenum with absence of the third and fourth portions, as well as absence of the superior mesenteric artery and jejunum apple peel atresia.
Sebben G. et al. (2013)	45 cadavers	Cadaveric dissection	Among the 45 cadavers analyzed, 7 presented anatomical variations related to SMA. In three cases, the right hepatic artery originated from SMA, corresponding to 10% of the sample. In two of the cadavers, the SMA originated the common hepatic artery. It was also found a single case of the left hepatic artery arising from SMA and one case of SMA originating the gastroduodenal artery.
Taha et al. (2017)	A Sudanese male cadaver	Cadaveric dissection	SMA was observed by forming an arch over the confluence of the inferior vena cava and left renal vein. Other variations were found: 1) The SMA shared the same origin as the celiac trunk; 2) The unusual origin of the right hepatic artery.
Torres et al. (1999)	34-week newborn with intestinal atresia	Ultrasonographyand Abdominal Radiography	Ultrasound examination of the abdomen suggested the absence of SMA shortly after its removal from the abdominal aorta and with hypertrophy of the celiac axis. The two distal thirds of the transverse, descending and rectosigmoid colon were present. No calcifications were observed in the abdominal cavity, as occasionally occurs with intestinal atresia.
Weber and Freeman (1999)	36-week newborn with duodenojejunal atresia	Laparotomy	There was loss of the third and fourth parts of the duodenum due to the absence of the SMA branch. The distal segment of the ileum is shortened and assumes the helical configuration around a retrograde perfusion vessel, which compensates for the missing SMA. This case involved complete obliteration of SMA together with associated duodenal atresia.
Wu Y et al. (2014)	A 69-year-old woman	Computed tomography	The study revealed the complete absence of SMA and compensatory dilation of the inferior mesenteric artery. The aneurysm of the splenic artery and the inferior phrenic arteries that emerged aberrantly from the aorta on the same level of the celiac trunk were also observed.
Yakura et al. (2017)	An 86-year-old female cadaver	Cadavericdissection	SMA originated the cystic artery. The middle colonic artery was absent and the left colonic artery, branching from the inferior mesenteric artery, was distributed along the entire length of the transverse colon.
Yoo et al. (2011)	An 82-year-old Korean female cadaver	Cadavericdissection	SMA gave the inferior mesenteric artery as its second branch. The longitudinal vessels of the anastomosis between the superior mesenteric artery and the inferior mesenteric artery survived to form the common mesenteric artery.

**FIGURE 2:**
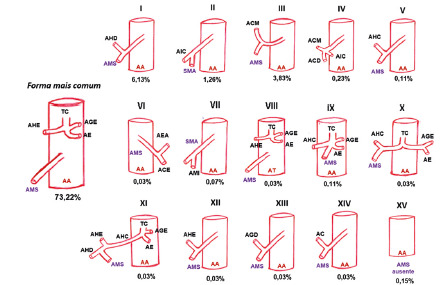
SMA in its most common origin to the left. In right, SMA variant forms.

The most common form of the emergence of SMA and its variants, direct or indirect, found in the analysis of the selected works totaled 16 forms and were represented in Figure 2 for a better understanding. The central vascular axis represents the abdominal segment of the aortic artery, except in the variant form VIII whose trunk represents the thoracic aorta artery.

LHA=left hepatic artery; LCA=left colic artery; SMA=superior mesenteric artery; ASA=accessory splenic artery; IMA=inferior artery mesenteric; RHA=right hepatic artery; ICA=ileocolic artery; CA=cistic artery; MCA=colic artery media; GDA=gastroduodenal artery; RCA=right colic artery; SA=splenic artery; AA=aorta artery; AT=thoracic artery; CHA=common hepatic artery; CT=celiac trunk

## DISCUSSION

This study proposes a review on the anatomical variations involving the SMA, from its origin variant aspects, as to its variant aspects about which vessel it may originate. It also relates these variant aspects and verifies the anatomical variations of SMA as well as its clinical and surgical implications, performed through different methods.

During the embryonic development, there is the formation of four ventral splenic vessels, where after its maturation two central roots disappear, thus, remaining the first and fourth roots that originate the anastomoses of the celiac trunk and SMA. If there is a bifurcation between these arteries at a level different than normal, there may be displacement of some vessel from the celiac trunk to SMA^20^, generating the possibility of variations involving vessels of origin or destination of the celiac trunk and the SMA^6^.

In a study with 45 cadavers, seven cases were identified with anatomical variations related to SMA. In two, it was originated the common hepatic artery; in one case the left hepatic artery; and in three, the right hepatic artery originated, the latter being the most significant variation presented in this study^22^. This finding is consistent with the ones of Farghadani et al.^2^, that evaluated 607 patients by computed tomography, observing 219 (36.1%) individuals with some type of variation, the most common being the SMA originated from the right hepatic artery, present in 9.6% of cases. This type of variation is highly relevant both for its higher prevalence and for its potential risk during procedures in the area, since this condition exposes these vessels to suffer damages during surgical approaches involving this region.

It was also verified in the studies of Fonseca Neto et al.^3^, that SMA originated the right hepatic artery. Of the 479 patients who underwent liver transplantations, 63 (13.15%) had some type of SMA variation. Of those, 27 presented SMA originating the right hepatic artery, while the other four presented right hepatic artery from SMA, as well as the left hepatic artery originating from the left gastric artery. In this context, the detailed knowledge of these variations in SMA involving hepatic arterial anatomy is of great interest to surgeons who develop procedures in this area, especially liver transplants, since besides representing an ideal opportunity for their anatomical surgical study, their identification and correct handling are fundamental for the good outcome of the procedure^1^ ^,^ ^4^ ^,^ ^22^ ^,^ ^29^.

Another relevant finding in the included studies in this review was the origin of SMA and celiac trunk from the thoracic aorta, 9 mm and 21 mm above the aortic hiatus, respectively. The SMA trajectory descends at the thoracoabdominal level, inducing the formation of a 17º angle, having a 9 mm aortomesenteric distance at the level of the lower duodenum. For this reason, the patient would be likely to simultaneously develop a triple syndrome: the celiac axis compression syndrome, that is, compression of the celiac trunk by the median arcuate ligament, SMA compression syndrome (SMA compression by median arcuate ligament), and SMA syndrome (duodenum compression by SMA)^16^.

The SMA was present in 100% of the sample in the studies of Olave et al.^18^, in which more than 50% of the sample observed the SMA at the L1 level, these findings may serve as a morphological support for the surgical procedures that involve the management of abdominal organs, especially the posterior ones.

SMA is known to supply the middle intestinal loop of the primitive intestine that originates the distal half of the duodenum, 3^rd^ and 4^th^ duodenal parts, jejunum, ileum, cecum and vermiform appendix, ascending colon and 2/3 of the transverse colon. It is possible that the SMA is absent and in these cases other vessels may supply some of these structures, on the other hand, it is also possible that areas of this primitive intestine are without vascularization which may lead to atresia or a delay in intestinal development preventing its normal function. 

Saša et al.^21^ revealed a case of a 29-week-old premature infant who did not have SMA, and consequently did not develop the distal part of the duodenum as well as the jejunum, undergoing surgery to remove the atresic portion ligating the functional ends. Another study also observed, in a 34-week-old child, SMA absence, and consequently absence of the jejunum, ileum, cecum and appendix as well as the ascending colon and the proximal part of the transverse colon^24^. The compensatory hypertrophy of the celiac trunk kept part of the duodenum extension. Weber and Freeman^25^ found atresia of the distal duodenum in a 36-week-old child due to absence of the inferior duodenal pancreatic branch (SMA branch).

Variations were also observed regarding the SMA absence in adults, accompanied by compensatory dilation of the inferior mesenteric artery. Acknowledging this issue is essential for health professionals, especially for medical surgeons who perform rectal and sigmoid colon surgeries, because in these cases, ligation of the inferior mesenteric artery during these procedures would bring harmful consequences to the subject, since in such situations, the inferior mesenteric would be the only artery responsible to supply the structures derived from the middle and posterior intestine^26^. In addition, the SMA absence in adults is rare, but in newborns it is reported as the cause of congenital duodenojejunal atresia, which contributes to defects in the formation and absorption of the entire median intestine, since irrigation of this area is dependent on this vessel. Congenital atresia and duodenal stenosis are often responsible for intestinal obstructions, occurring in 1:5,000-10,000 live births and affect males more than females^10^.

Classical SMA arises as a collateral branch, anterior to the abdominal aorta artery. As to its variant forms, there was a greater predominance of SMA originating the right hepatic artery.

Current surgical procedures, including transplants, vascular reconstructions as well as abdominal surgeries, require detailed technical knowledge about the regional vascular anatomy, being of fundamental importance for the success of the procedure. Knowledge about the possibility of non-existence of SMA has an influence on the development of important clinical and surgical conditions, such as duodenojejunal atresia in newborns, and surgeons who perform liver transplantation, allowing professionals to plan and conduct better their treatment interventions appropriately.

## CONCLUSIONS

SMA variations are not uncommon findings and their reports evidenced through the scientific literature demonstrate a great role for the development of important clinical conditions, making knowledge about this subject relevant to surgeons and professionals working in this area.
